# Factors associated with mobile medical clinic use: a retrospective cohort study

**DOI:** 10.1186/s12939-023-02004-3

**Published:** 2023-09-26

**Authors:** Angela Coaston, Soo-Jeong Lee, Julene K. Johnson, Sandra Weiss, Thomas Hoffmann, Caroline Stephens

**Affiliations:** 1 California Baptist University, College of Nursing, 8432 Magnolia Ave, Riverside, Ca 92504 USA; 2 University of California, San Francisco, CA 94143 USA; 3 University of Utah, Salt Lake City, UT 84112 USA

**Keywords:** Mobile clinics, Utilization, Access to health care, Chronic disease, Andersen’s behavioral model

## Abstract

**Background:**

Mobile medical clinics have been used for decades to provide primary and preventive care to underserved populations. While several studies have examined their return on investment and impact on chronic disease management outcomes in the Mid-Atlantic and East Coast regions of the United States, little is known about the characteristics and clinical outcomes of adults who receive care aboard mobile clinics on the West Coast region. Guided by the Anderson Behavioral Model, this study describes the predisposing, enabling, and need factors associated with mobile medical clinic use among mobile medical clinic patients in Southern California and examines the relationship between mobile clinic utilization and presence and control of diabetes and hypertension.

**Methods:**

We conducted a retrospective cohort study of 411 adults who received care in four mobile clinic locations in Southern California from January 1, 2018, to December 31, 2019. Data were collected from patient charts on predisposing (e.g., sex, race, age), enabling (e.g., insurance and housing status), and need (e.g., chronic illness) factors based on Andersen’s Behavioral Model. Zero-truncated negative binomial regression was used to examine the association of chronic illness (hypertension and diabetes) with number of clinic visits, accounting for potential confounding factors.

**Results:**

Over the course of the 2-year study period, 411 patients made 1790 visits to the mobile medical clinic. The majority of patients were female (68%), Hispanic (78%), married (47%), with a mean age of 50 (SD = 11). Forty-four percent had hypertension and 29% had diabetes. Frequency of mobile clinic utilization was significantly associated with chronic illness. Patients with hypertension and diabetes had 1.22 and 1.61 times the rate of mobile medical clinic visit than those without those conditions, respectively (IRR = 1.61, 95% CI, 1.36–1.92; 1.22, 95% CI, 1.02–1.45).

**Conclusions:**

Mobile clinics serve as an important system of health care delivery, especially for adults with uncontrolled diabetes and hypertension.

## Background

Mobile medical clinics are custom-made vehicles (e.g., vans, trucks, recreational vehicles) converted to healthcare clinics that travel to the heart of communities where people work, live, play, and pray [1]. Over the past few decades, mobile medical clinics have contributed to the health of underserved populations who are marginalized by geographic, social, or structural barriers [2, 3] by providing primary care, preventative care, and health care screening [4]. They are an effective strategy for increasing access to healthcare and reducing health disparities for communities [3]. In addition, an increasing body of knowledge shows that mobile medical clinics are accomplishing their mission to provide cost-effective healthcare for underserved populations across the nation [3, 5].

Foundational studies on mobile medical clinics have been conducted in the Mid-Atlantic and east coast regions of the United States (US). These studies evaluated the effectiveness of mobile medical clinics by calculating and investigating their return on investment, [6–9] evaluating chronic disease management outcomes, [3, 5–10], and identifying utilization patterns [2]. Results suggest that mobile medical clinics are a model for high-quality, cost-effective health care delivery for improving health outcomes in underserved areas. However, variations in region, populations, and healthcare service patterns across the nation underscore the need to understand how mobile medical clinics impact healthcare access, healthcare utilization, health outcomes, and cost. Although there are a reported 120 mobile health clinics providing services in California, with 70 licensed by the state as a primary care clinic, [11] no studies to date have described the demographic characteristics, healthcare utilization patterns, and chronic illness (e.g., diabetes, hypertension) health outcomes of adults served by primary care mobile medical clinics operating in the western region of the US.

Applying theoretical models of health services utilization to underserved populations, such as mobile clinic patients can be particularly helpful in identifying the unique challenges they face in obtaining needed services and maintaining or improving their health status [12]. Andersen’s Behavioral Model is one of the most frequently used frameworks for explaining and predicting patient utilization of healthcare services and related outcomes [13–15]. Guided by Andersen’s Behavioral Model, this study examined the characteristics, mobile clinic utilization, and clinical outcomes of adults who receive care at Well of Healing Mobile Medical Clinic, which is a no-cost, faith-based primary care mobile medical clinic in Southern California [16].

The aims of this study were to: 1) describe visit frequency among adults who utilize mobile clinics by sociodemographic and health characteristics (i.e., age, sex, ethnicity, insurance status, zip code, Charlson Comorbidity Index score) and presence and control of chronic illness (i.e., diabetes: hemoglobin A1c < 6.5% and hypertension: blood pressure < 140/90 mmHg); and 2) examine the relationship between mobile clinic utilization and presence of chronic illness, controlling for sociodemographic and health characteristics.

## Methods

### Study design and sampling

This study was a retrospective cohort study analyzing data retrieved from chart reviews of adult patients who visited four mobile clinic locations in Southern California from January 1, 2018, to December 31, 2019. The study inclusion criteria included adults 26 years and older who visited the clinic at least one time during the 2-year study period. As chronic illnesses were the outcome of interest, this study included only adult patients over age 26 – an approach consistent with other mobile clinic studies [13, 17]. Exclusion criteria included those who visited the clinic once but left without being seen by a health care provider, and people 25 years and younger. After conducting an initial patient chart review, a total of 425 patients were identified as eligible for the study. Upon further review, 14 patients were excluded from the study because they left without being seen by a provider. Hence, 411 patients were included in the study.

### Study setting

San Bernardino County is one of the largest counties in California. Many areas suffer from poverty-related disparities, including food deserts, high rates of crime, unemployment, and homelessness [18]. San Bernardino County residents experience higher levels of diabetes and hypertension compared to residents in the rest of the state of California (diabetes: 11% vs 9%; hypertension: 32% vs 28%) [18]. Additionally, 36% of adults in San Bernardino County are obese, compared with California's obesity rate of 27.9% [18].

The Well of Healing Mobile Medical Clinic is a primary care clinic that opened its doors in 2004. It is licensed as a free clinic by the California Department of Public Health for San Bernardino County. It is a non-denominational faith-based clinic operated by ministry volunteer physicians and nurses [16]. The mobile clinic drives into four communities: Fontana, Ontario, Muscoy, and San Bernardino. Clinic services are offered once a month at each of the clinic locations. For continuity of care, patients and volunteers are assigned to one of the four sites. The sites are set up at a local church in each of these communities. Patients are served on a first come, first serve basis; no one is turned away. First, patients are assessed by a nurse. Then, they are given a private examination by a provider, either a physician, physician assistant, or nurse practitioner. This examination may be followed by lab draw, medication prescription when appropriate, and health education.

### Study variables and measures

The dependent variable was mobile clinic utilization. Mobile clinic utilization was operationally defined as the total number of visits per year during the 2-year study period and the mean number of visits per year.

The independent variables were categorized as predisposing, enabling, and need factors following Andersen’s Behavioral Model. Predisposing factors refer to the propensity of individuals to use services [19] and included demographic characteristics including sex (male or female), race/ethnicity (African American, White, or Hispanic), age, and marital status (single or married). Enabling factors refer to resources specific to the individual or attributes of the community in which the person lives [19] that either enable or impede healthcare utilization. Such enabling factors for this study included insurance status (uninsured, insured, and other) and housing status (housed or homeless based on having a zip code). Need factors refer to the level of illness which is the most proximate cause of health service use and may be either perceived by the individual or identified by a care provider [19]. Such need factors included: the presence of obesity and/or depression; the Charlson Comorbidity Index (CCI) score; and chronic illness diagnoses of diabetes and hypertension, and their control status based on hemoglobin A1c level and systolic and diastolic blood pressure, respectively.

The presence of obesity and depression at baseline were ascertained by chart review of a diagnosis of obesity [20] or depression, [21] or an existing medication prescription for depression (Abilify, Elavil, Prozac, or Zoloft based on known available mobile clinic treatment options). While it is possible that these prescriptions for depression could be used for other mental health disorders (e.g., anxiety, panic, post-traumatic stress disorders, Schizophrenia, etc.), chart review did not reveal these other mental health diagnoses. The Charlson Comorbidity Index (CCI) documents the presence of 19 comorbidities (e.g., age, CHF, CVA, COPD), allocates a weight of 1–6 based upon the adjusted relative risk of 1-year mortality, and is then summed to provide a total index score which serves as an indicator of disease burden and a strong estimation of mortality [22–24]. Based on distribution of CCI scores in the study sample, the CCI variable was coded categorically (CCI = 0, 1, 2, 3, 4 +), with CCI = 0 as the referent category.

Presence of diabetes was identified in the patient chart by the record of the disease diagnosis and/or presence of diabetes medication (e.g., metformin, glipizide). Controlled diabetes was defined as hemoglobin A1c < 6.5%, based on the Centers for Disease Control and Prevention (CDC) guidelines [25]. Hemoglobin A1c provides an average level of blood sugar over the past 3 months. The higher the Hemoglobin A1c level, the more poorly diabetes is controlled [26]. Hemoglobin A1c tests were extracted from all visits in which hemoglobin A1c was tested.

The presence of hypertension was identified in the patient chart by the record of the disease diagnosis and/or presence of hypertensive medication (e.g., Amlodipine, HCTZ, Losartan, Lisinopril). All systolic and diastolic blood pressure recorded values were extracted from each patient record. Controlled hypertension was defined as systolic pressure < 140 mmHg and diastolic pressure < 90 mmHg. According to JNC-8 hypertension guidelines, American Academy of Family Physicians, and the setting’s standard of practice, for individuals 18 to 59 years of age without major comorbidities and patients 60 years or older who have diabetes, chronic kidney disease (CKD), or both conditions, the blood pressure goal is < 140/90 mmHg [27, 28].

### Data analyses

Descriptive statistics were used to summarize the characteristics of the sample and the total number of visits by sample characteristics. Descriptive statistics were reported as means and standard deviations for continuous variables and frequencies and percentages for categorical variables. We examined the relationship between the number of mobile clinic visits and the presence of chronic illness (diabetes and hypertension) using both poisson and negative binomial regressions and conducted a formal test of poisson versus negative binomial on the full multivariable model to determine a better model. Since individuals were required to have at least one visit to the clinic in order to be considered part of the cohort and the counts were overdispersed, we chose a zero-truncated negative binomial regression.

Covariates of predisposing, enabling, and need factors were included based on a theoretical approach. The analysis adjusted for an exposure time offset which was calculated for members who were already in the mobile clinic health care system from 01/01/2018 (study start) to 12/31/2019 (study end) and for new patients who started to visit during the study time period, from the time of their first visit to 12/31/2019 (study end). Unadjusted and adjusted results were presented with Incident Rate Ratios (IRR) and 95% Confidence Intervals (CIs). Data analysis was conducted in R v4.1.0 [29] using the R package VGAM v1.1.5 [30].

## Results

### Characteristics of overall study sample

Table 1 presents the baseline characteristics, total number of clinic visits, and mean number of visits for adults who received care at a Southern California mobile medical clinic between January 1, 2018, and December 31, 2019. Over the course of the 2-year study period, 411 patients made a total of 1790 mobile medical clinic visits. The majority of the mobile clinic patients were female (*n* = 281, 68%), Hispanic (*n* = 321, 78%), and married (*n* = 192, 47%), with a mean age of 50 (range 42–57, SD = 11). Uninsured individuals accounted for 38% (*n* = 156) of the study sample. Nearly all mobile clinic patients reported a home address (*n* = 402, 98%) while only 2% (*n* = 9) indicated homeless housing status.

**Table 1:** Baseline characteristics and clinic visits of patients receiving care at mobile medical clinics in Southern California between January 1, 2018, and December 31, 2019

Variables	Mobile clinic patients^a^ n (%)	Total number of visits^b^ n	Number of clinic visits^c^ mean (SD)
TOTAL SAMPLE^d^	411 (100.0)	1790	4.4 (3.9)
Initial visit before 2018	158 (38.4)		1.7 (1.4)
Initial visit 2018–2019	253 (61.6)		2.7 (2.5)
**Predisposing variables**			
Gender/Sex			
Female	281 (68.4)	1229	4.4 (3.9)
Male	130 (31.6)	561	4.3 (4.0)
Race/Ethnicity			
Hispanic	321 (78.1)	1513	4.7 (4.0)
African American	25 (6.1)	63	2.5 (3.3)
White	24 (5.8)	67	2.8 (3.5)
Unspecified	41 (10.0)	147	3.6 (3.2)
Age			
26–35	29 (7.1)	53	1.8 (1.6)
36 -45	107 (26.0)	329	3.5 (3.4)
46–55	141 (34.3)	568	4.3 (3.9)
56–65	93 (22.6)	577	5.3 (4.2)
66 +	37 (9.0)	263	5.4 (4.3)
Mean (SD)	50.3 (11.2)		6.6 (4.2)
Marital Status			
Married	192 (46.7)	511	5.0 (4.1)
Single	151 (36.7)	957	3.4 (3.5)
Unknown	68 (16.5)	322	4.7 (3.8)
**Enabling variables**			
Insurance Status			
Uninsured	156 (38.0)	934	6.0 (4.3)
Unknown	230 (56.0)	75	3.4 (3.3)
Other	25 (6.1)	781	3.0 (3.2)
Housing Status			
Housed	402 (97.8)	1137	4.4 (3.9)
Homeless	9 (2.2)	776	1.1 (0.3)
Clinic Location			
Ontario, CA	160 (38.9)	533	4.6 (4.1)
Fontana, CA	119 (29.0)	61	5.2 (3.8)
Muscoy, CA	99 (24.1)	413	3.6 (3.9)
San Bernardino, CA	33 (8.0)	474	2.3 (2.7)
**Need variables**		472	
Chronic Illness			
Hypertension	181 (44.0)	1137	6.3 (4.3)
Uncontrolled hypertension	89 (49.2)	627	7.0 (4.2)
Diabetes	117 (28.5)	776	6.6 (4.2)
Uncontrolled diabetes	69 (59.0)	602	8.7 (3.7)
Other Medical Conditions			
Obesity	89 (21.7)	533	6.0 (4.5)
Depression	10 (2.4)	61	6.1 (6.3)
Charlson Comorbidity Index score			
0	150 (36.5)	413	2.8 (2.6)
1	105 (25.6)	474	4.5 (4.0)
2	84 (20.4)	472	5.6 (4.4)
3	50 (12.2)	328	6.6 (4.3)
4 +	22 (5.4)	103	4.7 (4.2)

^a^Mobile clinic patients = number of patients included in the study who visited the clinic between January 1, 2018, and December 31, 2019

^b^Total number of visits = number of visits during the study period between January 1, 2018—December 31, 2019

^c^Mean number of visits over the 2-year study period with standard deviation. SD = Standard Deviation

^d^Row Percent

Of the total 411 mobile clinic patients, 181 (44%) had hypertension and 117 (29%) had diabetes. The mean number of visits for those with hypertension (n = 181) and uncontrolled hypertension (n = 89) were 6.3 (SD = 4.3) and 7.0 (SD = 4.2), respectively. The mean number of visits for those with diabetes (n = 117) and uncontrolled diabetes (n = 69)were 6.6 (SD = 4.2) and 8.7 (SD = 3.7), respectively. Those with depression accounted for 2.4% of the study sample (*n* = 10) and on average, these patients visited mobile clinics 6.1 (SD = 6.3) times over the study period. Roughly two-thirds of patients (*n* = 261, 63%) had some level of comorbidity (CCI = 1–4 +). Mobile clinic patients with a CCI score of 3 had two times the mean number of visits than those with a CCI score of 0 [CCI 3: 6.6 (SD = 4.3); CCI 0: 2.8 (SD = 2.6)].

### Characteristics of patients with diabetes and uncontrolled diabetes

Table 2 presents baseline characteristics of the patients with a diagnosis of diabetes and uncontrolled diabetes. Among the mobile clinic patients with diabetes (*n* = 117, 29%), 68% (*n* = 80) were female, 82% (*n* = 92) were Hispanic, 55% (*n* = 67) were married, 51% (*n* = 60) were uninsured, 100% (*n* = 117) were housed, and their mean age was 55.5 (SD = 10.0) years old. Of those with diabetes, 70% (*n* = 82) also had a diagnosis of hypertension.

**Table 2:** Baseline characteristics of patients with diabetes and uncontrolled diabetes receiving care at a mobile medical clinics in Southern California between January 1, 2018, and December 31, 2019

**Variables**	**Diabetes Yes**^**a**^	**Uncontrolled**^**b**^ Hemoglobin **A1c ≥ 6.5**
TOTAL SAMPLE*	117 (28.5)	69 (59.0)
**Predisposing variables**		
Gender/Sex		
Female	80 (68.4)	47 (68.1)
Male	37 (31.6)	22 (31.9)
Race/Ethnicity		
Hispanic	96 (82.1)	57 (82.6)
African American	3 (2.6)	1 (1.4)
White	6 (5.1)	3 (4.3)
Unspecified	12 (10.3)	8 (11.6)
Age		
25–35	1 (0.9)	0 (0.0)
36 -45	22 (18.8)	14 (20.3)
46–55	28 (23.9)	13 (18.8)
56–65	49 (41.9)	31 (44.9)
66 +	17 (14.5)	11 (15.9)
Mean (SD)	55.54 (10.0)	56.41(10.3)
Marital Status		
Married	67 (57.3)	39 (56.5)
Single	28 (23.9)	14 (20.3)
Unknown	22 (18.8)	16 (23.2)
**Enabling variables**		
Insurance Status		
Uninsured	60 (51.3)	41 (59.4)
Unknown	53 (45.3)	25 (36.2)
Other	4 (3.4)	3 (4.3)
Housing Status		
Housed	117 (100.0)	69 (100.0)
Homeless	0 (0.0)	0 (0.0)
Clinic Location		
Ontario	46 (39.3)	28 (40.6)
Fontana	44 (37.6)	29 (42.0)
Muscoy	20 (17.1)	9 (13.0)
San Bernardino	7 (6.0)	3 (4.3)
**Need variables**		
Chronic Illness		
Hypertension	82 (70.1)	50 (72.5)
Diabetes	117 (100.0)	69 (100.0)
Other Medical Conditions		
Obesity	32 (27.4)	21 (30.4)
Depression	1 (0.9)	1 (1.4)
Charlson Comorbidity Index Score		
0	9 (7.7)	2 (2.9)
1	30 (25.6)	18 (26.1)
2	37 (31.6)	23 (33.3)
3	30 (25.6)	20 (29.0)
4 +	11 (9.4)	6 (8.7)

^a^Diabetes Yes = Had a written diagnosis of diabetes in the chart, recorded hemoglobin A1c > 6.5, or was taking diabetes medication (i.e., metformin, glipizide)

^b^Diabetes Uncontrolled = hemoglobin A1c > 6.5 at the start of the study

^*^Row Percent

Among those with diabetes, 59% had uncontrolled diabetes (*n* = 69) as measured by hemoglobin A1c > 6.5%. The majority of mobile clinic patients with uncontrolled diabetes were female (*n* = 47, 68%), Hispanic (*n* = 57, 83%), and uninsured (*n* = 41, 60%) with comorbid hypertension (*n* = 50, 73%) and obesity (*n* = 21, 30%).

### Characteristics of patients with hypertension and uncontrolled hypertension

Table 3 presents baseline characteristics of the patients at the mobile medical clinic with a diagnosis of hypertension and uncontrolled hypertension. Of the 411 patients in the study, 44% (*n* = 181) had a diagnosis of hypertension, of these, 49% (*n* = 89) had uncontrolled hypertension (systolic blood pressure > 140 and/or diastolic blood pressure > 90).

**Table 3:** Baseline characteristics of patients with hypertension and uncontrolled hypertension receiving care at mobile medical clinics in Southern California between January 1, 2018, and December 31, 2019

Variables	Hypertension Yes^a^	Uncontrolled^b^ systolic > 140 or diastolic ≥ 90 mmHg
TOTAL SAMPLE*	181(44.0)	89 (49.2)
**Predisposing variables**		
Gender/Sex		
Female	125 (69.1)	61 (68.5)
Male	56 (30.9)	28 (31.5)
Race/Ethnicity		
Hispanic	148 (81.8)	74 (83.1)
African American	10 (5.5)	7 (7.9)
White	7 (3.9)	1 (1.1)
Unspecified	16 (8.8)	7 (7.9)
Age		
Mean (SD)	57.36 (9.9)	56.48 (9.1)
Marital Status		
Married	102 (56.4)	53 (59.6)
Single	53 (29.3)	24 (27.0)
Unknown	26 (14.4)	12 (13.5)
**Enabling variables**		
Insurance Status		
Uninsured	91 (50.3)	53 (59.6)
Unknown	78 (43.1)	32 (36.0)
Insured	12 (6.6)	4 (4.5)
Housing Status		
Housed	179 (98.9)	89 (100.0)
Homeless	2 (1.1)	0 (0.0)
Clinic Location		
Ontario	63 (34.8)	30 (33.7)
Fontana	62 (34.3)	29 (32.6)
Muscoy	42 (23.2)	23 (25.8)
San Bernardino	14 (7.7)	7 (7.9)
**Need variables**		
Chronic Illness		
Hypertension	181 (100.0)	89 (100.0)
Diabetes	82 (45.3)	42 (47.2)
Other Medical Conditions		
Obesity	50 (27.6)	22 (24.7)
Depression	4 (2.2)	2 (2.2)
Charlson Comorbidity Index Score		
0	33 (18.2)	18 (20.2)
1	46 (25.4)	24 (27.0)
2	57 (31.5)	29 (32.6)
3	35 (19.3)	15 (16.9)
4 +	10 (5.5)	3 (3.4)

^a^Hypertension Yes = Had a written diagnosis of Hypertension in the chart, Systolic > 140 and/or Diastolic > 90

^b^Hypertension Uncontrolled = record of blood pressure > 140/90 start of study

^*^Row Percent

Among mobile clinic patients with hypertension, 69% were female (*n* = 125), 82% Hispanic (*n* = 148), 56% married (*n* = 102), 50% uninsured (*n* = 91) and 99% housed (*n* = 179) with a mean age of 57.3 (SD = 9.9). Nearly half of these individuals (*n* = 89, 49%) had uncontrolled hypertension (Systolic > 140/Diastolic > 90). Of those with uncontrolled hypertension, the majority were female (*n* = 61, 68%), Hispanic (*n* = 74, 83%), and uninsured (*n* = 53, 60%) with 47% having comorbid diabetes (*n* = 42), 25% obesity (*n* = 22), and 2% depression (*n* = 2).

### Rates of mobile medical clinic visits

Table 4 presents the univariate and the multivariate relationships between rates of mobile medical clinic visits and patient characteristics and chronic illness.

**Table 4:** Zero truncated negative binomial regression: rates of mobile medical clinic visits per year by chronic illness in a sample of mobile medical clinic patients between January 1, 2018—December 31, 2019 (*N* = 411)

Variable	Unadjusted model IRR (95% CI)	Adjusted model: IRR (95% CI)
All		
**Predisposing variables**		
Gender/Sex		
Female	1.04 (0.84–1.28)	1.02 (.09–1.20)
Male	Referent	Referent
Race/Ethnicity		
Hispanic	2.09 (1.34–3.26) ****	1.68 (1.14–2.48) ***
African American	1.02 (0.54–1.93)	1.07 (0.62–1.87)
White	Referent	Referent
Unspecified	1.35 (0.80–2.28)	1.38 (0.87–2.21)
Age		
25–35	Referent	Referent
36 -45	2.37 (1.34–4.19) ***	1.42 (0.87–2.33)
46–55	2.99 (1.71–5.22) ****	1.32 (0.80–2.19)
56–65	3.80 (2.17–6.65) ****	1.12 (0.64–1.96)
66 +	3.36 (1.87–6.05) ****	0.98 (0.54–1.79)
Marital Status		
Single	0.63(0.51–0.78) ****	0.74(0.62–0.89) ****
Married	Referent	Referent
Unknown	0.77 (0.60- 0.99) **	0.95 (0.77–1.17)
**Enabling variables**		
Insurance Status		
Uninsured	2.10 (1.37–3.24) ****	1.40 (0.96–2.06)
Other	Referent	Referent
Unknown	1.09 (0.71–1.67)	0.94 (.64–1.38)
**Need variables**		
Chronic Illness		
Hypertension	2.31 (1.93–2.76) ****	1.61 (1.36–1.92) ****
Diabetes	1.89 (1.56–2.29) ****	1.22 (1.02–1.45) *
Other Medical Conditions		
Obesity	1.52 (1.22–1.90) ****	1.27 (1.07–1.51) ****
Depression	1.32 (2.34–2.59)	–
Charlson Comorbidity index Score		
0	Referent	Referent
1	1.91 (1.49–2.45) ****	1.55 (1.22–1.98) ****
2	2.21 (1.72–2.85) ****	1.74 (1.26–2.40) ****
3	2.46 (1.85–3.27) ****	1.88 (1.27–2.77) ***
4 +	1.69 (1.13–2.54) **	1.54 (0.95–2.50)

*Abbreviations: IRR* Incident Rate Ratio, *CI* Confidence Interval

*Significance levels:* *p < .05, **p < .01, ***p < .001, ****p < .0001

^d^The adjusted model included all variables except for depression because the cell number was below 5

In the unadjusted model, race/ethnicity was significantly associated with the frequency of mobile clinic utilization, with Hispanics having almost 2 times the rate of visits as Whites (IRR = 2.09, 95% CI, 1.34–3.26). Age was also significant, showing higher rates of visits among ages 36–66 + . Those who were single had significantly higher rates of visits compared to those who were married (IRR = 0.63, 95% CI, 0.51–0.78).

In the adjusted model, race/ethnicity remained significantly associated with the rate of mobile clinic utilization, with Hispanics having almost 2 times the rate of visits than Whites (IRR = 1.68, 95% CI, 1.14–2.48). Those who were single had significantly higher rates of visits compared to those who were married (IRR = 0.74, 95% CI, 0.62–0.89). Age, insurance status, and a CCI score of 4 were no longer significant after adjusting for sociodemographic and health characteristics.

Chronic illness need variables were all significant factors in mobile clinic utilization. Patients with hypertension had 1.61 times the rate of mobile clinic visits than those without hypertension (IRR = 1.61, 95% CI, 1.36–1.92). Mobile clinic patients with diabetes had 1.22 times the rate of a mobile medical clinic visits than those without diabetes (IRR = 1.22, 95% CI, 1.02–1.45). Those with obesity had 1.27 times the rate of visits than those without obesity (IRR = 1.27, 95% CI, 1.07–1.51). In addition, individuals with a CCI score of 1, 2, and 3 had 1.55, 1.74, and 1.88 times the rate of mobile clinic visits than those with a CCI score of 0 (IRR = 1.55, 95% CI, 1.22–1.98), (IRR = 1.74, 95% CI,1.26–2.40), (IRR = 1.88, 95% CI, 1.27–2.77), respectively.

## Discussion

To our knowledge, this is the first mobile medical clinic study of the western region of the US and the first to use the Andersen Behavioral Model to describe mobile medical clinic patient characteristics and factors associated with mobile clinic utilization in this region. This retrospective cohort study described the predisposing, enabling, and need factors associated with mobile medical clinic use among mobile medical clinic patients in Southern California. It further examined the relationship between mobile clinic utilization and presence and control of diabetes and hypertension. We hypothesized that patients with chronic illness (diabetes and hypertension) will have a higher rates of mobile clinic visits compared to those without chronic illness, controlling for sociodemographic and health characteristics. Study findings revealed that patients with both hypertension and diabetes had higher rates of mobile clinic visits than those without those chronic conditions.

Applying a theoretical model of health services utilization, like the Anderson Behavioral Model, to underserved mobile clinic populations, can help elucidate some of the unique challenges faced in obtaining the services required to maintain and improve health status [12]. For example, this study demonstrated that mobile clinics intervene in particular demographics: married Hispanic females, between the ages of 46–57, living with some level of chronic illness burden (e.g., CCI score of 1–4), and insurance status as either not reported or uninsured. These findings are consistent with other national studies, [10, 17, 31] with the main exception of race/ethnicity. This study had a much higher proportion of mobile clinic patients who were Hispanic (78%) compared to other studies that ranged from 19%—35% [10, 31, 32]. This is reflective of the western region of the US and the areas’ overall demographic, potentially offering unique insights into possible access to care challenges for this population [33].

In terms of mobile clinic utilization, the patients in this cohort had fairly similar mean visits per year compared to other studies [34] (4.4–7.0 vs 2.5–6.9, respectively). Consistent with the literature and the Andersen Behavioral Model, this study demonstrated that rates of mobile clinic utilization depended mainly on predisposing factors such as ethnicity and need factors such as chronic illness status [35]. Surprisingly, enabling factors (i.e., insurance status) were not associated with mobile medical clinic use.

Notably, individuals who were Hispanic visited the Southern California mobile clinics at almost 2 times the rate of Whites. Such a finding highlights the importance for geographic variability in clinical delivery and research studies [36]. Ultimately, chronic illness diagnosis and burden (CCI score 1–4) had the greatest influence on frequency of mobile clinic visits. Such need characteristics are most proximal to service use and prompt the importance for medical care [13, 19]. Compared to other studies [17, 32], this mobile clinic population had much higher prevalence rates of hypertension (44%) and diabetes (29%), as well as significant uncontrolled disease. Moreover, this study further found that those with hypertension and diabetes had higher mobile clinic visit rates than those without such chronic illnesses. Mobile clinics provide a unique opportunity to deliver care to underserved individuals with chronic illness by addressing social determinants of health; delivering timely high-quality health care services; reducing travel distance; and eliminating transportation issues and scheduling challenges for historically marginalized individuals and communities [33].

### Limitations

This study has some important limitations to consider. First, this study was a secondary analysis of retrospective clinical chart data that was not originally collected for the purposes of research. As a result, some demographic and clinical visit data were not consistently recorded by clinic personnel. For example, some demographic information was either not reported or recorded as unknown. For example, transportation, an important social determinant of health, was not provided on the chart information [1]. In addition, there was limited chart information on education and income – two enabling variables known to influence mobile clinic utilization [2, 10, 32].

Second, the clinic was staffed by volunteer providers which introduced a potential discrepancy in standard of care and systematic data collection. For example, a hemoglobin A1c may be ordered by a physician after an exam every 3 months, while another may request a hemoglobin A1c every 6 months, thus there were varying dates and frequency of hemoglobin A1c readings and BP readings, and some patients had more readings than others. Consequently, when assessing associations between variables and the number of visits per year, some individuals received fewer follow ups than others. We adjusted for this by using the standard offset adjustment in the zero truncated negative binomial regression analysis.

Finally, this study was also limited in its access to medical records from traditional settings including the local Health Information Exchange. This data would provide insight on whether patients seek care only at the mobile medical clinic or if they were seeking care elsewhere. Literature shows mobile clinic users may use various sources in seeking medical care. (Gibson et al., 2014). Nevertheless, we believe that this study is valuable in its current presentation of characteristics, frequency of visits, and associations between mobile medical clinic visit rates despite the potential of other health care use options.

### Implications for practice, policy, and research

From a practice standpoint, the results of this study can guide considerations of sociodemographic characteristics in practice at mobile medical clinics (e.g., age, gender, ethnicity). Knowledge of gendered demographics in visiting mobile clinics creates opportunities for gender-focused interventions. For example, by knowing that more women than men visit this clinic, the clinic could use these findings to develop new programs for women’s health screenings and education.

Given the higher visit rates among patients with chronic illness, specialty services should be considered for these patients including endocrinology, cardiology, and podiatry. With nearly half of the mobile clinic population having chronic illness, specialists could help stave off the progression of disease and improve care outcomes using their specialized approach to care. Telehealth could be used on the clinic for these specialty services, minimizing cost to the specialist, while providing a much-needed service to the underserved individuals.

With the high prevalence of hypertension, diabetes, and obesity, emphasizing lifestyle modification interventions, such as diet recommendations, stress management, and exercise, is essential to improve healthcare outcomes effectively. These non-pharmaceutical interventions would be in combination with pharmaceutical interventions. In addition, this data could be used for developing grants to support new programs that target a specific population in need.

From a policy standpoint, access to care is essential for disease prevention and promotion of good health. Policy makers can leverage the knowledge gained from this study to promote the utilization of mobile medical clinics as a consistent source of care for underserved groups. Further, policy makers can advocate for government agencies serving the disenfranchised and minority groups that lack healthcare access due to transportation, lack of funds, or lack of insurance.

From a research perspective, additional work is needed to improve our understanding of other factors, such as education level and income that may influence use of mobile medical clinics. In the future, prospective studies would be useful to ensure that data is standardized and collected at specific time-points for measurement over time. Standards of care should be followed, such that each patient has a set of biomedical readings during the same time frame and with the same time interval.

Overall, this study expands our understanding of the characteristics of individuals who receive care aboard mobile medical clinics, particularly in the Western region of the U.S. In particular, our study contributes data on adults with insurance and chronic illness who visit mobile clinics. Care should be taken to locate mobile clinics close to the community most in need, as bridging gaps in health delivery offers to improve chronic illness, decrease emergency room utilization, and increase regular source of care [33]. Future studies are needed to examine how mobile clinics can be integrated with health systems to decrease readmissions of patients with chronic illnesses such as diabetes, hypertension, chronic heart failure, and chronic kidney disease. Mobile medical clinics could be a downstream solution for these patients who are often readmitted due to an unmet need for care located close to home and provided by trusted sources [33]. 

## Conclusions

In the present study, individuals’ characteristics and use of mobile medical clinics in Southern California differed as a function of the presence and control of chronic illness. In describing the factors associated with mobile medical clinic use, the present study demonstrated associations between clinic utilization and presence and control of chronic illness. This study contributes to a growing body of evidence that mobile clinics serve as an important system of health care delivery, especially to underserved populations, those who are both insured and uninsured, and those with uncontrolled chronic illness. Regardless of insurance status, access to mobile medical clinics in the areas were people, work, live, pray, and play is essential to increasing their use of services.

The deployment of mobile medical clinics in communities of underserved individuals is a step toward improving healthcare access including high quality, cost-effective care for patients. Understanding factors associated with mobile medical clinic utilization informs providers, policy makers, health care leaders, and government health care representatives with evidence on alternative ways to bridge the gap in health inequities, recognize the value of mobile medical clinics, and invest in mobile medical clinics, thereby addressing social determinants of health and improving chronic illness health outcomes for all who utilize them. Given the present study’s findings, public health, health systems, universities, and private organizations can leverage the descriptive characteristics of patients who visit clinics to guide policy in support of these clinic modalities, guide practice interventions specific to gender and chronic illness needs, guide research for improving health delivery models, and promote philanthropic and grant funding for these community-focused services.

## Data Availability

The data that support the findings of this study are available on request from the corresponding author. The data are not publicly available due to privacy or ethical restrictions.
